# Feasibility of three dimensional and strain transthoracic echocardiography in a single-centre dedicated NHS cardio-oncology clinic

**DOI:** 10.1186/s44156-024-00063-y

**Published:** 2024-12-16

**Authors:** Patrick O’Driscoll, David Gent, Liam Corbett, Rod Stables, Rebecca Dobson

**Affiliations:** 1 Liverpool Heart and Chest Hospital NHS Foundation Trust, Liverpool, UK; 2 Liverpool Centre for Cardiovascular Science, Liverpool, UK; 3 University of Liverpool, Liverpool, UK

**Keywords:** Three-dimensional echocardiography, Global longitudinal strain, Cardio-oncology, Left ventricular ejection fraction, Transthoracic echocardiography, Feasibility

## Abstract

**Background:**

Following the publication of international cardio-oncology (CO) imaging guidelines, standard echocardiographic monitoring parameters of left ventricular systolic function have been endorsed. Recommendations highlight that either two-dimensional (2D) or three-dimensional (3D) left ventricular ejection fraction (LVEF), alongside global longitudinal strain (GLS) should be routinely performed for surveillance of patients at risk of cancer therapy-related cardiac dysfunction (CTRCD). We studied the feasibility of 3D-LVEF, 2D-GLS and 2D-LVEF in a dedicated CO service.

**Methods:**

This was a single-centre prospective analysis of consecutive all-comer patients (n = 105) referred to an NHS CO clinic. Using a dedicated Philips EPIQ CVx v7.0, with X5-1 3D-transducer and 3DQA software, we sought to acquire and analyse 2D- and 3D-LVEF and 2D-GLS, adhering to the British Society of Echocardiography (BSE) and British Cardio-Oncology Society (BCOS) transthoracic echocardiography protocol.

**Results:**

A total of 105 patients were enrolled in the study; 5 were excluded due to carcinoid heart disease (n = 5). Calculation of 3D-LVEF was achieved in 40% (n = 40), 2D-GLS in 73% (n = 73), and 2D-LVEF in 81% (n = 81). LV quantification was not possible in 19% (n = 19) due to poor myocardial border definition. Strong correlation existed between 2D-LVEF and 3D-LVEF (r = 0.94, p < 0.0001). Bland–Altman plot demonstrated no statistical differences in that the mean deviation between 2D-LVEF and 3D-LVEF were consistent throughout a range of LVEF values. The most persistent obstacle to 3D-LVEF acquisition was insufficient myocardial border tracking (n = 30, 50%).

**Conclusion:**

This study demonstrates the high feasibility of 2D-GLS and 2D-LVEF, even in those with challenging echocardiographic windows. The lower feasibility of 3D-LVEF limits its real-world clinical application, even though only a small difference in agreement with 2D-LVEF calculation was found when successfully performed.

## Background

We are currently in an era of remarkable growth of dedicated cardio-oncology (CO) services. With demographic change, an aging population is associated with an increasing incidence of cancer alongside being more likely to have associated co-morbidities, including cardiovascular disease [1] or cardiovascular risk factors. Modern systemic anti-cancer therapies (SACT) include a range of agents with the potential for cardiotoxicity. Cardiovascular complications of SACT can be immediate or delayed and include valve disease, pericardial disease, and left ventricular (LV) systolic dysfunction. Transthoracic echocardiography (TTE) remains the cornerstone imaging modality for accurate and reproducible assessment of cardiac structure and function within CO services [2].

It is widely accepted that a reduced or borderline low LV ejection fraction (LVEF) of 50–54% detected before commencing anti- cancer therapies identifies patients at an increased risk of developing cancer therapy-related cardiotoxicity (CTRCD) [3]. Risk stratification, including baseline cardiac function assessment is therefore essential to define cardiac status and assess whether CTRCD has taken place during and/or after treatment. Novel developments in TTE function quantification now enable clinicians to potentially diagnose CTRCD at a sub-clinical stage. Although in most cases SACT can continue, clinically significant parameters identified by these more novel TTE indices permit a larger window of opportunity for timely intervention that may help in reducing the risk of disruption to the oncological treatment pathway.

To ensure TTE examinations are timely, robust, reproducible, and systematic, guidance has been released by the British Society of Echocardiography (BSE) and the British Cardio-Oncology Society (BCOS) to serve as a blueprint for CO services [4]. Central to the document is the recommendation that LV function should be quantified by advanced TTE imaging techniques, namely three-dimensional (3D) calculation of LV volumes, LVEF and global longitudinal strain (2D-GLS). These novel TTE indices are not without challenges. For instance, the ability of 3D echocardiography to overcome geometric assumptions of 2D Simpson’s biplane is counterbalanced by a reduction in temporal resolution [5], whilst the high test-re-test reproducibility of 2D-GLS is reliant on optimal 2D images before reliable analysis can take place [6]. We sought to determine the feasibility of performing and analysing 3D-LVEF, 2D-GLS, and 2D-LVEF in a dedicated NHS CO clinic in accordance with current recommendations [4].

## Methods

### Patient selection

A total of 105 consecutive patients attending the Liverpool Heart and Chest Hospital CO clinic between October 2021 and April 2022 were prospectively enrolled for inclusion in this single-centre analysis. Exclusion criteria were patients with carcinoid heart disease as they undergo a scan adhering to a separate protocol. No restriction on a previous diagnosis of cancer, ischaemic heart disease, valvular heart disease, atrial fibrillation, breast, and lung surgery was applied. Systolic function of the LV was assessed where feasible as part of a complete echocardiogram in accordance with BSE/BCOS CO dataset guidance [4].

### Equipment

Transthoracic echocardiography was performed on a Philips EPIQ CVx v7.0 ultrasound machine with the X5-1 transducer, and analysis conducted on-cart using Philips TOMTEC LV Autostrain for 2D-GLS analysis and 3DQA software to calculate LV volumes and LVEF. The TTE examinations were performed by one of three highly experienced echocardiographers.

### Echocardiography

Patients had a full TTE performed according to the BSE minimum dataset [7] with additional images and analysis as outlined in the BSE/BCOS CO guidelines [4]. No contrast/ultrasound enhancing agents were used for patients with poor echocardiographic windows, which were defined according to the 2014 ASE recommendation as "the inability to detect two or more contiguous segments in any three of the apical windows” [8]. These patients were instead referred for cardiac magnetic resonance imaging (cMRI) for LV function quantification.

### Quantification of LV systolic function

#### 3D LV ejection fraction

Using Philips 3DQA software, a 3D full volume dataset was acquired from the apical four chamber window (A4C) and created by acquiring four beat sub-volumes over sequential cardiac cycles. Particular attention was paid to patient breath hold at end expiration during shallow breathing to avoid stitch artefact. The real-time electrocardiogram (ECG) trace was of high quality to ensure full volume triggering was appropriate and the region of interest (ROI) was seen within the scanning plane in all phases of the cardiac cycle. Image optimisation settings, frame rate and image depth were all adjusted to acquire the highest quality image. Before 3D acquisition was accepted, a quality review of the 2D (A4C) and apical two chamber (A2C) images were completed. Studies where the patient was in an arrhythmia e.g., atrial fibrillation (AF) were not absolutely contraindicated, however the increase in probability of stitch artefact from multiple sub-volumes was recognised and analysis deemed futile if present.

#### 2D Global longitudinal strain

Global longitudinal strain was measured by acquiring focused, high quality 2D images of the LV from the A4C, A2C, and apical three chamber (A3C) windows. The ECG signal was optimised with particular attention paid to heart rate variability across three cardiac cycles. A frame rate between 40 to 90 frames/sec at a heart rate of 50–100 bpm was maintained. Sector width and depth were appropriately adjusted as previously described. Having selected all three apical views to be analysed, TOMTEC LV AutoStrain software calculated 2D-GLS of the left ventricular myocardium as a percentage change from its original length, providing a negative (−) value. Visual assessment of appropriate border tracking for each view ensured that automated tracking was accurate, if two segments or more were not adequately tracked after minimal manual adjustment, then 2D-GLS was discarded.

#### 2D Simpson’s biplane method

On cart analysis of 2D-LVEF Simpson’s biplane method was performed by acquiring images of the LV from the A2C and A4C windows. Acquisition was made after adjusting machine settings as previously highlighted, to ensure optimal endocardial border definition and avoid image foreshortening. The endocardial/blood border was traced at end-diastole and end-systole in each of the two views, with papillary muscles and trabeculations included as part of the LV chamber volume. Length of the LV was extrapolated as the distance between the midpoint of the mitral valve annulus line to the most distal point of the LV apex. Examinations where two or more contiguous segments in a single view were not visualised were excluded from analysis.

### Statistical analysis

Statistical analysis was performed by Stats Direct v3.35 (Wirral, UK). Categorical variables are presented as counts and percentages. Continuous variables are presented as mean  ±  standard deviation. Bland–Altman plot was used to investigate the relationship between 3D-LVEF and 2D-LVEF measurements. Between group differences for frame rate and multi-beats were compared using an unpaired t*-*test, with a two-sided P value of < 0.05 considered to indicate statistical significance. Pearson’s correlation coefficient was used to compare LV function assessment quantification between 2D-LVEF and 3D-LVEF techniques.

## Results

**Fig. 1:**
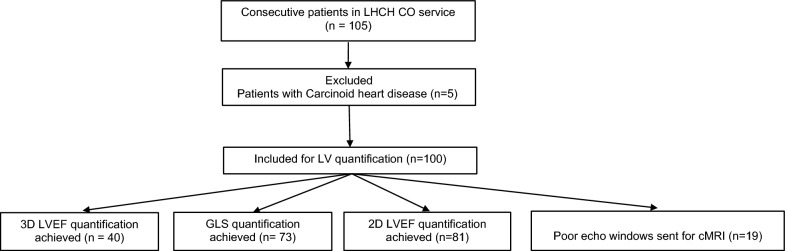
Patient enrolment and analysis

A total of 105 consecutive patients were enrolled into the study between October 2021 and April 2022. After exclusion criteria were applied, 100 patients were eligible for analysis (Fig. 1). Patient baseline demographics are summarised in Table 1 with patient physiological characteristics grouped by LV function quantification method in Table 2. Image quality was high enough to enable assessment of 3D-LVEF in 40% (n = 40), 2D-GLS in 73% (n = 73) and 2D-LVEF in 81% (n = 81). No quantification was possible for 19% (n = 19), as we were unable to detect two or more contiguous segments in any three of the apical windows.

**Table 1:** Patient baseline demographics pre-echocardiogram

	Total (n = 100)	Male (n = 51, 51%)	Female (n = 49, 49%)
Age (years)	64 ± 12	67 ± 11	61 ± 12
Body surface area (m^2^)	1.91 ± 0.24	2.02 ± 0.21	1.79 ± 0.22
Systolic blood pressure (mmHg)	142 ± 21	140 ± 20	144 ± 22
Diastolic blood pressure (mmHg)	83 ± 13	80 ± 13	86 ± 13
Heart rate (bpm)	82 ± 17	81 ± 18	83 ± 16

Gender is expressed as n (%); all other data are expressed as mean and ± standard deviation

**Table 2:** Physiological characteristics of the patient and the method of LV function quantification

	2D-LVEF	3D-LVEF	GLS	No quantification
Number of patients	81 (81%)	40 (40%)	73 (73%)	19 (19%)
Female sex, number of patients (%)	45 (55%)	21 (53%)	41 (56%)	4 (4%)
BSA, (m^2^)	1.87 ± 0.22	1.84 ± 0.2	1.87 ± 0.21	2.09 ± 0.23
Heart rate, bpm	82 ± 16	78.3 ± 15	79.8 ± 14	81 ± 19
Systolic blood pressure, mmHg	141 ± 22	143 ± 22	142 ± 22.4	143 ± 17
Diastolic blood pressure, mmHg	82 ± 13	82.3 ± 13	82 ± 13.2	84 ± 13
Sinus rhythm, number of patients (%)	71 (88%)	36 (90%)	65 (89%)	19 (100%)

Gender is expressed as n (%); all other data are expressed as mean and ± standard deviation

The most persistent barriers to LV quantification (Table 3) were insufficient myocardial border tracking with 30 (50%) of 60 failed attempts for 3D-LVEF, 12 (44%) from the 27 failed attempts for 2D-GLS and 8 (42%) from the 19 failed attempts for 2D-LVEF.

**Table 3:** Barriers to acquiring full BSE recommended LV quantification dataset

Obstacle to analysis	3D LVEF	2D GLS	2D LVEF
Raised BSA (> 2 m^2^ male, 1.6 m^2^ female)	14	7	7
Insufficient myocardial border tracking	30	12	8
Low BSA (< 1.7 m^2^ male, < 1.6 m^2^ female)	4	2	1
Septal dyssynchrony	2	2	1
Arrhythmia (AV block or frequent ectopy)	5	1	0
Post-surgical dressings in situ	2	1	1
Technical error (poor ECG signal, incomplete acquisition)	2	1	0
Chest wall deformity (pectus excavatum or carinatum)	1	1	1
Total	60	27	19

**Fig. 2:**
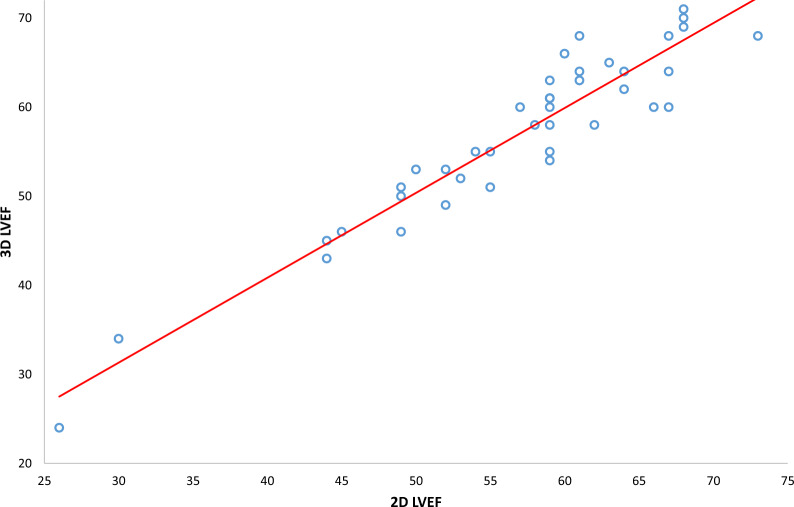
Pearson’s correlation coefficient plot comparing left ventricular ejection fraction (LVEF) as calculated by 3D-LVEF versus standard 2D Simpson’s biplane method

**Fig. 3:**
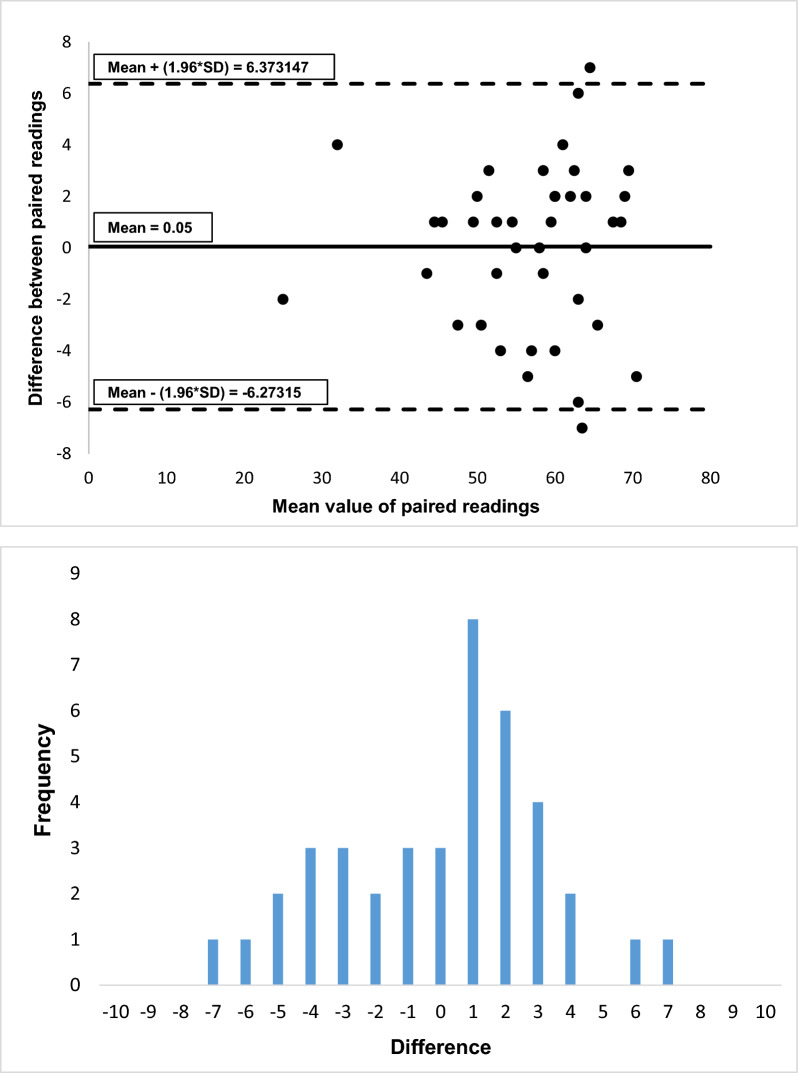
Bland–Altman plot for 3D-LVEF and 2D-LVEF calculation agreement and frequency distribution histogram

There was strong correlation in the LVEF calculated by 3D and 2D echocardiography (Fig. 2, r = 0.94, p =  < 0.0001). Bland–Altman plots for agreement between 3D-LVEF and 2D-LVEF calculation, and frequency distribution histogram are shown in Fig. 3, No statistical differences were shown between 2D-LVEF and 3D-LVEF, throughout a range of LVEF values (P = 0.05, SD:3.22, 95% CI = − 6.273 to 6.373).

For the 81 patients with quantifiable 2D-LVEF images, frame rate (Hz) was documented for 67 patients. Of this group, 40 were successfully analysed by 3D with a mean frame rate of 47 ± 19 Hz and a mean multi-beat acquisition of 3.6 beats ± 1. For the 27 (40%) where 3D was unsuccessful, a mean frame rate of 42 ± 22 Hz with a mean multi-beat acquisition of 3.5 beats ± 1. The between group differences for frame rate and multi-beats were p = 0.35 and p = 0.58 respectively.

## Discussion

CO guidance strongly recommends 3D-LVEF and 2D-GLS measurement when monitoring cardiac function in patients at risk of CTRCD to identify cardiac dysfunction at the earliest stage [4]. Central to this is the ability to acquire good quality echocardiographic images. Our prospective real-world analysis of an unselected CO cohort demonstrated that 3D-LVEF assessment is less feasible than 2D-GLS and 2D-LVEF assessment; when 3D-LVEF was feasible, there was little variation compared to 2D-LVEF across a range of ejection fractions. In most cases where 2D-LVEF measurement could be performed, 2D-GLS was feasible. The most common factors precluding measurement of any LV function quantification were insufficient myocardial border tracking (n = 8, 42%) and raised body surface area (BSA) (n = 7, 37%). This finding is not unique to our study [9, 10] and is a well-recognised TTE disadvantage. Overall, our study shares the optimism for the capability of 2D-GLS to improve TTE sensitivity to diagnose CTRCD at a sub-clinical stage [11].

### Two-dimensional LVEF Simpson’s biplane method

Left ventricular volume and 2D-LVEF estimation by Simpson’s biplane is fundamental in cardiac function quantification and therefore diagnosis of CTRCD [4, 12, 13]. In our study, 2D-LVEF was more feasible than 3D-LVEF and 2D-GLS. However, there are recognised limitations with using biplane assessments, including inaccuracies with respect to non-geometric ventricles and apex foreshortening, that are overcome with 3D-LVEF analysis [5, 14–16]. In our study, where both techniques were feasible, there was strong agreement between 3D-LVEF and 2D-LVEF, with mean differences between 2D-LVEF and 3D-LVEF small and consistent throughout a range of LVEF values (r = 0.94, p < 0.0001). Practical differences in techniques cannot be ignored as 2D-LVEF Simpson’s method was performed manually compared to semi-automated border tracking with 2D-GLS. This factor may account for a difference in their feasibility. It has also been shown that 2D-LVEF exhibits greater inter- and intra-operator variability across serial TTE scans, which may be higher than the thresholds that define cardiotoxicity [4, 17]. This limitation is of clinical importance as treatment may or may not be initiated based on operator variability rather than a reduction in LV function.

### Three-dimensional LVEF

3D-LVEF allows accurate serial quantification of LVEF with low test–retest inter- and intra-operator variability [18] and the measured LVEF is more comparable to gold standard cMRI [14]. The precision of semi-automated 3D-LVEF analysis packages will enable clinicians to make confident treatment decisions that reflect real interval changes in LV function, rather than operator variability [17].

As mentioned above, when 3D-LVEF was feasible, the measurements were in close agreement to those acquired by 2D-LVEF assessment. However, only 40% (n = 40) of our patients were suitable for 3D-LVEF. This was primarily due to insufficient myocardial border tracking (n = 30, 50%) and/or raised BSA (n = 14, 23%). Our findings were comparable with current literature, which has reported successful 3D-LVEF in 66% of their pre-selected patient cohorts [9, 10].

The frame rate of 3DTTE can be as little as 25% of 2D TTE, making the trade-off between temporal and spatial resolution critical. To overcome this, we utilised the full volume multi-beat acquisition function. Despite an adequate mean frame rate of 42 ± 22 Hz across 3.5 ± 1 beats, and rejecting sub-volumes with stitch artefact, image quality was still deemed insufficient for accurate and reproducible 3D-LVEF calculation in a sizable proportion of patients (n = 27/67, 40%). There was no significant difference between the frame rate and multi-beat acquired in those individuals who 3D-LVEF could be calculated and those for whom it could not. This reinforces the importance of optimal patient characteristics in 3D TTE data acquisition.

We did not screen our patient population pre TTE to avoid selection bias and to reflect real-world practice. This may have resulted in a higher recruitment of individuals with poor echocardiographic windows, i.e., the inability to detect two or more contiguous segments in any three of the apical windows [8]. This contrasts with Thavendiranathan et al. [18], who did select patients based on 3D-LVEF image suitability, which may account for their finding that 3D TTE evaluation was feasible in a higher proportion (71%) of their study group.

### Two-dimensional global longitudinal strain

Two-dimensional 2D-GLS is an established method for measuring sub-clinical reduction in LV function. It also has a role in heart failure prognostication in the CO populations, both during and after cancer treatment [9, 19, 20]. This is of critical importance because, irrespective of symptomatology and baseline LVEF, early pharmacological intervention can prevent irreversible LV dysfunction [21].

The feasibility of performing 2D-GLS in our study was high, 90% of patients in whom 2D-LVEF was possible also had adequate imaging for 2D-GLS assessment. Similar findings have been reported previously and reinforce the utility of 2D-GLS in improving the sensitivity of CTRCD diagnosis, alongside LVEF measurement [9].

However, akin to 2D and 3D LVEF assessment, the principal barrier to 2D-GLS quantification was insufficient myocardial border tracking (2D-GLS: n = 12, 44%, 3D-LVEF: n = 30, 50%). This is irrespective of higher temporal and spatial resolution (2D-GLS: ~ 80 Hz vs 3D-LVEF: ~ 42 Hz). Measuring 2D-GLS requires less user input compared to 3D-LVEF which will increase its successful adoption into a user-friendly and timely analysis workflow. Encouragingly, echocardiographer competency level does not have a large impact on successful acquisition of 2D-GLS [22], however high quality 2D images are fundamental to ensure high quality, reproducible and accurate advanced imaging [9].

### Contrast enhanced echo in the CO population

The cancer population presents a technical challenge to echocardiographers due to the greater prevalence of endocardial border drop out, as a result of an increased incidence of surgical intervention (mastectomy or pneumonectomy), chemotherapy treatment and/or chest irradiation [23]. The use of contrast enhancing agents for greater LV opacification in patients with poor echocardiographic windows has become common practice for many echo labs. However, BSE/BCOS highlight data to suggest variability in serial CO TTE exams [17] and it is for this reason we did not utilise this technique. 8/19 excluded patient’s had poor echo windows, i.e. they were off axis or foreshortened, which we judged unable to be improved with contrast. For this cohort, we were able to refer on to cMRI for LV assessment.

### Study limitations

While our investigation reflects clinical practice, there are important limitations to note. Firstly, 3D TTE datasets could not be obtained for all patients due largely to poor image quality as participants were not screened for image quality. Additionally, we did not explore the underlying cancer diagnosis and its effects, if any, on image quality. The study may lack external validity as it took place in a single centre and although TTE studies were performed by experienced echocardiographers, we did not address test–retest variability, with respect to inter- and/or intra- observer variability. We were also unable to compare our data against cMRI, as this imaging modality is only requested if the TTE is equivocal, or image quality impedes analysis. Since our data collection began, there have been advancements in clinically available machine software and hardware upgrades which potentially would improve on our findings.

## Conclusion

A paucity of research exists comparing the feasibility of 3D-LVEF, 2D-GLS and 2D-LVEF TTE in CO clinics, with reference to the BSE/BCOS guidance. This is of importance as these techniques are recommended as the first line diagnostic tools in the latest clinical guidance for the CO population when monitoring cardiac function. Our study showed that 19% of patients referred to the CO clinic had echocardiographic windows that precluded any quantitative assessment of LV function. Of those scans of acceptable quality, 2D-LVEF and 2D-GLS measurement were feasible, but 3D-LVEF was only possible in 40%. The principal failure of 3D TTE was insufficient myocardial border tracking which may be improved by future technological advances in 3D technologies. Despite recent recommendations highlighting that all CO patients undergoing LVEF assessment should have a 3D TTE, this may not be a practical goal.

## Data Availability

The data underlying this article will be shared on reasonable request to the corresponding author.

## Abbreviations

2-D: Two-dimensional
3-D: Three-dimensional
2D-GLS: Two-dimensional global longitudinal strain
A4C: Apical four chamber
A3C: Apical three chamber
A2C: Apical two chamber
AF: Atrial fibrillation
BCOS: British Cardio-Oncology Society
BSA: Body surface area
BSE: British Society of Echocardiography
cMRI: Cardiac magnetic resonance imaging
CO: Cardio-oncology
CTRCD: Cancer therapy-related cardiac dysfunction
ECG: Electrocardiogram
LV: Left ventricle
LVEF: Left ventricular ejection fraction.
ROI: Region of interest
SACT: Systemic anti-cancer therapies
TTE: Transthoracic echocardiography
